# A rare association of emphysematous pyelonephritis with unrecognized diabetes and polycystic kidney

**DOI:** 10.4103/0971-4065.50676

**Published:** 2009-01

**Authors:** A. M. Azzini, P. Sette, G. Castellano, R. M. Dorizzi

**Affiliations:** Department of Infection Disease, University of Verona, 37134 G.B. Rossi Hospital, Verona, Italy; 1 Department of Intensive Care, G. Fracastoro Hospital, 37047, San Bonifacio (Verona), Italy; 2 Department of Chemical Biochemistry, Morgagni - Pierantoni Hospital, 47100, Forlì, Italy

**Keywords:** Emphysematous pyelonephritis (EPN), diabetes mellitus, polycystic kidney, dialysis

## Abstract

Emphysematous pyelonephritis (EPN) is a rare, severe, gas-forming infection for which the treatment of choice is often an immediate nephrectomy, although many reports exist of conservative treatment of cases with antibiotic therapy and percutaneous drainage of abscesses. It usually occurs in diabetic patients and less frequently in subjects with an obstruction of the corresponding renoureteral unit; other predisposing factors are not common. We report here the case of a 51 year-old woman with a rare association of unrecognized diabetes and bilateral polycystic kidney disease who developed monolateral EPN. She had an emergency right nephrectomy and was admitted to Intensive Care Unit (ICU) for septic shock after surgery, requiring intensive resuscitation. The patient was managed with Coupled Plasma Filtration Adsorption (CPFA). Her clinical conditions rapidly improved and the hemofiltration was soon suspended. Urine and blood cultures were positive for the same *Escherichia coli*, which was susceptible to all tested antibiotics. The patient was transferred to the Nephrology Division and was discharged from the hospital without further dialysis after 34 days. This case report is somewhat unique because of the unusual association between undetected diabetes and polycystic kidney as predisposing factors of a severe infection of the urinary tract.

## Introduction

Emphysematous pyelonephritis (EPN) is a rare, severe, gas-forming infection for which the treatment of choice is often an immediate nephrectomy, although many reports exist of conservative treatment of cases with antibiotic therapy and percutaneous drainage of abscesses. It usually occurs in diabetic patients and less frequently in subjects with an obstruction of the corresponding renoureteral unit; other predisposing factors are not common.

We report here the case of a woman with a rare association of unrecognized diabetes and bilateral polycystic kidney disease who developed monolateral EPN.

## Case Report

A 51 year-old female was admitted to the Emergency Department with a week's history of lethargy, asthenia, and vomiting. Her medical history did not reveal any pathology or pharmacological treatment.

**Figure 1:**
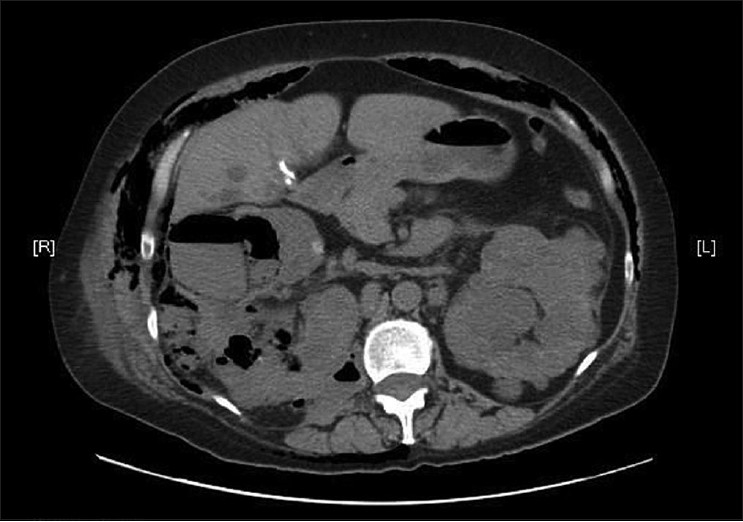
EPN on CT of abdomen; the subcutaneous emphysema extending from the abdomen's right side involves the thorax

At the time of admission, she was conscious and her laboratory investigations showed serum creatinine concentration = 3.14 mg/dL and glucose = 1044 mg/dL associated with severe polyuria, polydipsia, and leukocytosis (24 × 10^3^/L). An immediate diagnosis of diabetes was postulated. Physical examination was unremarkable with the exception of a wide subcutaneous emphysema involving the whole abdominal surface and extending up to the lower thorax. A contrast-enhanced chest-abdomen CT scan showed extensive retroperitoneal and extraperitoneal air, with bubbly gas collected in the kidney and under the abdominal right flank muscles. Both the kidneys had a polycystic structure, the right one was larger than the left, and fluid material was detectable in all the cysts [Figure 1].

As the patient's condition rapidly worsened, an emergency right nephrectomy was performed and broad-spectrum antibiotics (piperacillin-tazobactam) were empirically started. She was admitted immediately after surgery to the intensive care unit (ICU) for septic shock, requiring intensive resuscitation. The patient needed coupled plasma filtration adsorption (CPFA) for four days as well and was extubated after four days; vasopressors were stopped and the dialysis was suspended due to improvement in renal function. Urine and blood cultures were positive for the same *E. coli*, which was susceptible to all the tested antibiotics. Table 1 shows the biochemical and hematological parameters monitored during the patient's ICU stay. The patient was transferred to the Nephrology Division on the sixth day and was discharged from the hospital without any further dialysis after 34 days.

**Table 1:** Biochemical and hematological parameter during patient's ICU stay

	Day 1	Day 2	Day 3	Day 4	Day 5	Day 6
Creatinine (mg/dL)	3.14	2.01*	2.73*	2.07*	1.98	1.95
Glucose (mg/dL)	1044	258	266	122	180	131
Leucocytes (x10^3^/L)	24.02	15.7	8.71	11.9	8.97	7.70
Daily urinary output (mL)	150	2464*	638*	4140*	3340	1650
Platelets (x10^3^/L)	62	80	86	92	110	145
Sodium (mmol/L)	109	140	139	139	142	140
Potassium (mmol/L)	4.64	3.91	4.06	3.5	3.8	4.1
CRP (mg/L)	269	259	248	156	79	58
PCT (ng/mL)	27.9	27.7	17.4	9.8	4.2	2.1

* CPFA treatment; ICU = Intensive care unit; CRP = C-reactive protein; PCT = Procalcitonin

## Discussion

EPN is a rare, necrotizing infection of the renal parenchyma and its surrounding areas, that produces gas into the collecting system or perinephric tissue.[1] It is much more common in females than males (ratio 5–6:1), and involves both kidneys in <10% of the cases.[1–3] It is a life-threatening infection with a mortality rate as high as 80%.[4]

A strong association has been reported between glucose disorders and EPN, and it is a common presentation of urinary tract infection in diabetic patients.[5–9] Diabetes increases hospital admissions for EPN by 20–30 fold for patients under the age of 45 and by 3–5 fold for those over 45 years of age.[5]

EPN also affects patients with obstruction of the corresponding renoureteral unit. The infecting organisms are usually glucose-fermenting bacteria, with *E. coli* (69%) and *Klebsiella pneumoniae* (29%) being the most common.[8] Bacteremia occurs in more than half of the cases.[3,8]

Both the mechanism of gas formation and the pathogenesis of EPN remain unclear: possible pathways are mixed acid fermentation of glucose by Enterobacteriaceae (*i.e. E. coli* and *K. pneumoniae*) *via* anaerobic butyric fermentation of glucose. Huang *et al*. postulated that the pathogenesis of EPN could be gas-forming bacteria, high tissue glucose levels, impaired tissue perfusion, or a defective immune response[10] (neutrophil function is impaired in the presence of high urinary or tissue glucose concentrations).[7] The hypoxic environment of the kidney medulla, especially in end-stage diabetic nephropathy with microvascular disease, could predispose these patients to tissue ischemia and necrosis, thus potentiating the growth of gas-forming microbes.

Imaging is essential to diagnose EPN and a CT scan of the abdomen is preferred as it allows quantitation of the amount of gas and of renal parenchyma destruction. It is important also to assess the severity of the disease and to choose the type of treatment for the patient: a combination of percutaneous drainage and antibiotics, or early nephrectomy. In this case, we utilized a new technique, the so-called CPFA. We chose this novel treatment because it simultaneously removes different inflammatory mediators (interleukins, cytokines, and plasma factors) *via* unselective adsorption by a highly hydrophobic synthetic resin and has been proposed for improving severe sepsis and for treating septic shock.[11,12] In this case, CPFA was performed with a 0.5 m^2^ plasma filter in series with a 0.7 m^2^ polysulfone hemodiafilter. The plasma filtrate was circulated through a sorbent cartridge (MediaSorb, Bellco, Mirandola, Italy) and reinfused in the extracorporeal circuit.

Wan *et al.*[1] and Huang *et al.*[10] proposed two different classifications based on the prognosis and on the amount of produced gas respectively.

Wan *et al.*[1] recognized two EPN types that had different prognostic outcomes. Type I EPN is characterized by parenchymal destruction with streaky or mottled gas collection, but no fluid accumulation. On the other hand, type II is characterized by bubbly or loculated gas within the parenchyma or collecting system with associated renal or perirenal fluid accumulation. Prognosis for type I EPN is considered to be poorer than that for type II and the mortality rates have been reported to be 69 and 18% respectively.

Huang *et al.*[10] classified EPN into four classes according to the radiological findings of the CT scan: gas is present only in the collecting system in class 1 and in the renal parenchyma without extension to the extrarenal space in class 2. Class 3 is divided into two types: gas or abscess extend in the 3A type to the perinephric space, and to the pararenal space in the 3B type. A solitary kidney with EPN or bilateral disease are present in class 4.

In conclusion, EPN is a severe infection of the upper urinary tract and is susceptible to both a conservative or debulking treatment depending on the degree of the kidneys' involvement. An early combination of medical and surgical management is the key to successful care of EPN.
